# What about TSH and Anti-Thyroid Antibodies in Patients with Autoimmune Thyroiditis and Celiac Disease Using a Gluten-Free Diet? A Systematic Review

**DOI:** 10.3390/nu14081681

**Published:** 2022-04-18

**Authors:** Sabrina Malandrini, Pierpaolo Trimboli, Gabriele Guzzaloni, Camilla Virili, Barbara Lucchini

**Affiliations:** 1 Department of Internal Medicine, Regional Hospital of Locarno, 6600 Locarno, Switzerland; 2 Clinic for Endocrinology, Ente Ospedaliero Cantonale (EOC), 6500 Bellinzona, Switzerland; pierpaolo.trimboli@eoc.ch (P.T.); gabriele.guzzaloni@eoc.ch (G.G.); barbara.lucchini@eoc.ch (B.L.); 3 Faculty of Biomedical Sciences, Università della Svizzera Italiana (USI), 6900 Lugano, Switzerland; 4 Department of Medico-Surgical Sciences and Biotechnologies, Sapienza University of Rome, 04100 Latina, Italy; camilla.virili@uniroma1.it

**Keywords:** thyroid hormones, thyroid antibodies, celiac disease, gluten-free diet, systematic review

## Abstract

The prevalence of celiac disease (CD) in patients with chronic autoimmune thyroiditis (CAIT) is estimated to be between 2 and 7.8%. A gluten-free diet (GFD) in patients with CD is suggested to have a beneficial effect on CAIT. Thus, the present systematic review was undertaken to achieve more robust evidence about the change in thyroid stimulating hormone (TSH) and thyroid-specific antibodies (T-Ab) levels obtained in CD patients following a GFD. A specific search strategy was planned. The last search was performed on March 2022. The following data were mainly searched for in order to be extracted: sample size, mean and/or median with standard deviation (SD), and error (SE), individually, of thyroid hormones and T-Ab at baseline and after GFD, and the duration of the study. The initial search retrieved 297 records and 6 articles met the inclusion criteria. In total, 50 patients with both CD and CAIT and 45 controls were reported. The effects of a GFD on the thyroid hormonal and immunological profile could be extracted only in a part of the studies. Two studies were case reports. A low risk of bias was observed. These findings advise further studies, ideally randomized, in order to better investigate the potential relationship between GFD and thyroid homeostasis. The level of evidence is not still sufficient to recommend GFD to patients with CAIT.

## 1. Introduction

Chronic autoimmune thyroiditis (CAIT) is the most common autoimmune disease worldwide and is characterized by a high prevalence in females [1]. CAIT is characterized by a peculiar ultrasound pattern, thyroid-specific autoantibodies (T-Ab), and a variable degree of thyroid functional impairment at the time of diagnosis. Epidemiological studies have supported that CAIT, in a percentage variable between 14 and 29% of cases, may be associated with further endocrine and non-endocrine autoimmune diseases [2,3]. Among the latter, the most frequent associations involve rheumatic and gastrointestinal autoimmune diseases. The prevalence of celiac disease (CD) in patients with CAIT is estimated to be between 2 and 7.8% [4]. The reasons underpinning the coexistence of CAIT and CD in the same patient may be different. In fact, beside a shared immunogenetic background [5], an interaction between anti-transglutaminase IgA auto-antibodies, thyroid follicles, and the matrix [6] has been described. The association may be further justified based on the low serum levels of selenium and vitamin D observed in celiac patients [7,8]. From a clinical standpoint, hypothyroid patients with concomitant CD need a higher dose of levothyroxine to achieve the on-target thyroid stimulating hormone (TSH) when compared to patients without CD [9].

Interestingly, a gluten-free diet (GFD) in patients with CD is suggested to have a beneficial effect on CAIT. In fact, some studies have suggested that a GFD inhibits the autoimmune process by reducing the concentration of T-Ab, and contributing to the withdrawal of pathological changes in the small intestine [10]. However, to date, strong evidence (i.e., meta-analysis) about the favourable role of a GFD on thyroid hormones and T-Ab value fluctuations is lacking. This evidence may not have a negligible role in the management of both CD and CAIT patients. In fact, as reported in a meta-analysis, the prevalence of thyroid disease in CD patients is significantly higher when compared with that in controls, thus suggesting that CD patients should be screened for thyroid disease [11].

Following the above clinical issue, and according to the above clinical question, the present systematic review was undertaken to achieve more robust evidence about the change in TSH and T-Ab levels obtained in CD patients using a GFD.

## 2. Methods

### 2.1. Conduction of Review

The systematic review was performed according to MOOSE (Meta-analysis Of Observational Studies in Epidemiology) [12].

### 2.2. Search Strategy

A five-step search strategy was planned, as follows: (1) sentinel studies were searched in PubMed, (2) both keywords and MeSH terms were identified, (3) PubMed and Cochrane databases were searched, (4) studies reporting efficacy of GFD in terms of thyroid laboratory tests and T-Ab were detected, and (5) references of included studies were screened to find further papers. Studies reporting data of TSH and T-Ab collected before and after following a GFD were initially included. Those studies that did not detail the results in terms of TSH and T-Ab were excluded. To enlarge the number of studies to the maximum possible, no sample size criteria were used to include studies and also small series could be initially included in the systematic review. In addition, any kind of study (i.e., retrospective or prospective, observational or interventional, randomized or not, and controlled or not) could be initially included. Studies with overlapping data were excluded. The last search was performed on 1 March 2022. Articles written in English were always included and those in other languages were included when appropriate. No publication-year restriction was applied. Two investigators (S.M. and B.L.) independently searched papers, screened both titles and abstracts, reviewed the full-texts, and selected articles for their inclusion.

### 2.3. Data Extraction

The following information was independently searched and extracted by two authors (S.M. and B.L.) from the included studies: authors and their country of origin, year of publication, study type, study design, number of patients, mean and/or median with standard deviation (SD) and error (SE), individually, of thyroid hormones and T-Ab at baseline and after GFD, and the duration of the study. Data were cross-checked between the two authors, while discordances were discussed with the other authors.

### 2.4. Study Quality Assessment

The risk of bias of the included studies was assessed by two raters (S.M. and B.L.) through the Quality Assessment of Diagnostic Accuracy Studies (QUADAS-2) tool for the following aspects: patient selection, index test, reference standard, flow, and timing. Accordingly, risk of bias and concerns about applicability were rated as low, high, or unclear [13].

## 3. Results

### 3.1. Articles Retrieved

**Figure 1:**
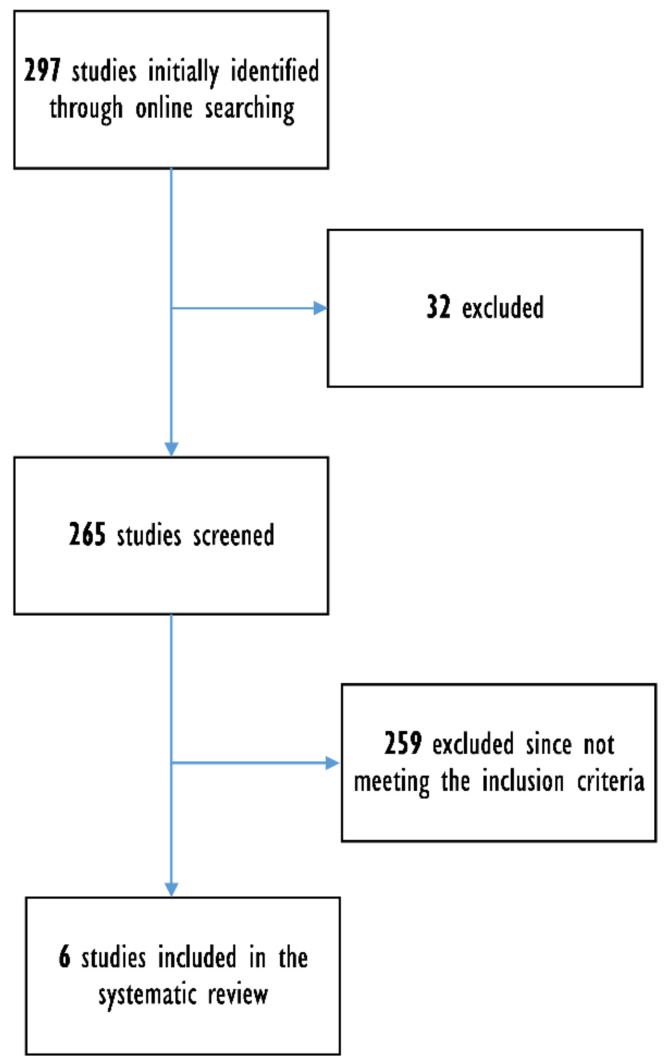
Flow of records.

The literature search process and the flow of records are reported in Figure 1. The initial search retrieved 297 records and, after screening them, six articles met the study inclusion criteria and were selected [14,15,16,17,18,19]. MOOSE checklist is reported in the Supplementary Materials Table S1.

### 3.2. Qualitative Analysis (Systematic Review)

As a whole, the study group included 50 patients with CD and CAIT and 45 controls. The characteristics of the studies are summarized in Table 1, while the effects of a GFD on the hormonal and immunological profile are illustrated in Table 2.

In the study by Metso [14], at baseline, patients and controls were matched by age and sex. Two patients did not maintain a strict adherence to a GFD. No statistical differences in TSH and fT4 levels between the gluten-free group and the control group at baseline and during the follow-up were found. In the study by Krysiak [15], at baseline, patients and controls were comparable with respect to age; body mass index; and serum levels of anti-thyroid peroxidase antibodies (TPO-Ab), anti-thyroglobulin antibodies (Tg-Ab), and thyroid hormones. The GFD was well tolerated and no patient dropped out of the study. The GFD reduced serum titres of TPO-Ab and Tg-Ab, as well as increased the serum levels of 25-hydroxyvitamin D, but did not affect significantly thyrotropin and free thyroid hormones. In the group with a normal diet, serum thyroid antigens, free thyroid hormones, TSH, and 25-hydroxyvitamin D remained at similar levels throughout the study.

In the case report by Asamoah [16], a GFD significantly reduced the gastrointestinal symptoms and, along with the switch to the L-T4 oral solution, enhanced the absorption of the thyroid hormone therapy and controlled the fluctuations in TSH levels.

In the study by Mainardi [17], in one patient, an initial increase in the antithyroid antibodies was observed. After 18 months, the patient had a euthyroid state with pharmacologic support and the antithyroid antibodies were decreased. The other patient initially refused a GFD and the serologic markers of CD and CAIT remained substantially unmodified. Six months later the patient accepted a GFD and was observed to have a persistent increase in TPO-Ab and Tg-Ab with subclinical hyperthyroidism development.

In the case report by Stramazzo [18], three months after starting a GFD, the TSH concentration was unchanged and the TPO-Ab and Tg-Ab remained elevated.

In the study by Valentino [19], the three patients showed an improvement of symptoms regarding to hypothyroidism, and a reduction in the hormonal replacement therapy was noted. No variations were observed in the TPO-Ab and Tg-Ab titres. Only one patient, with a longer follow-up of 18 months, revealed a significant decrease in TPO-Ab titre (from 2.580 ± 430 to 451 ± 18 UI/mL, *p* < 0.001; mean of three different determinations with standard deviations).

A control group was present in two studies. The controls used in the study by Krysiak [15] were woman aged between 20 and 45 years old with recently diagnosed and previously untreated autoimmune thyroiditis (i.e., with positive TPO-Ab, reduced echogenicity of the thyroid parenchyma on thyroid ultrasonography, and normal thyroid function), who were incidentally found to be positive for anti-tissue transglutaminase antibodies without clinical symptoms of CD. Importantly, the group encompassed patients who preferred to follow a gluten-containing diet. In the study by Metso [14], the control subjects were collected from patients of the outpatient clinic. They were suffering from cardiovascular disorders (hypertension, arrhythmia, or a suspicion of coronary artery disease), did not have clinical evidence of CD, and were following a gluten-containing diet.

### 3.3. Quality Assessment

The quality assessment of the studies is reported in Table 3. As a result of our rigorous selection criteria, a low risk of bias was recorded in all of the studies.

## 4. Discussion

The present systematic review was conceived to achieve evidence-based information about the improvement of serum TSH and T-Ab levels in CD patients when they follow a GFD. As the association between CAIT and CD is recognized, and considering the overall worldwide prevalence of CAIT, our study should have a significant impact in clinical practice.

Gluten is a molecule formed by glutenin polymers and gliadin monomers; both these proteins contain a high percentage of prolines and glutamines that protect them from complete degradation and digestion in the gastrointestinal tract [20]. In patients with CD, these gluten peptides trigger an inflammatory reaction also featured by the increased permeability of the intestinal barrier, a condition known as “leaky gut” [21]. This condition has been detected in several non-celiac autoimmune disorders, such as type 1 diabetes, Hashimoto’s thyroiditis, rheumatoid arthritis, and multiple sclerosis, to name but a few [22]. The increased permeability of the intestinal barrier may allow for the entrance of exogen compounds into systemic circulation [23]. Furthermore, it has been observed that gluten ingestion may affect gut microbiota composition [24], leading to a dysbiotic state that may enhance a vicious circle of gut epithelium damage; chronic inflammation; and, in people with a genetic predisposing background, autoimmunity [25].

Several studies in vitro and in animal models have hypothesized that gluten may impact key steps that may trigger autoimmunity, impairing both innate and/or adaptive immune branches. In non-obese diabetic (NOD) mice that are highly prone to spontaneously developing autoimmune disorders, a reduced expression and cytotoxicity of natural-killer cells toward a pancreatic beta cell line has been demonstrated [26]. Concerning the proinflammatory Th17 pathway, it appears to be increased in pancreatic lymph nodes of gluten-fed mice, but reduced in animals fed a gluten-free diet [27]. GFD exerts an anti-diabetic effect in the NOD murine model [28].

The literature about a possible positive effect of GFD on autoimmune disorders other than CD in human beings is large, but shows discrepant results. For example, a recent study on patients with concomitant CD and potential/subclinical pituitary autoimmunity suggests that GFD might be able to induce the remission of subclinical lymphocytic hypophysitis, or even prevent progression to a clinical stage [29]. On the other hand, a recent review article failed to find an impact of GFD on the prevention and prognosis of multiple sclerosis [30]. The data about a possible role of GFD in patients bearing both CAIT and CD are marred by different study designs and outcomes, such as the normalization of subclinical hypothyroidism [31], the variations of anti-thyroid antibodies [15,17], the normalization of thyroid volume [14], and the variations of thyroxine requirement in hypothyroid patients [9]. Furthermore, some of them are case reports or case series [16,17,18,19].

Our attempt to systematize the knowledge about this topic was aimed at quantifying the effect of GFD in CAIT patients, specifically examining the serum levels of TPO-Ab and Tg-Ab, as well as the levels of the more used marker of thyroid function, i.e., serum TSH values. Unfortunately, a meta-analysis could not be performed to estimate the variation of both TSH and T-Ab, as only six studies met our strict inclusion criteria, and the available data did not allow us to pool the findings from the six studies. Furthermore, the presented results do not reveal any effect of a GFD on the duration or severity of CAIT, also conflicting with the data regarding vitamin D absorption. Therefore, the level of evidence is not still sufficient to recommend a gluten-free diet to patients with CAIT, despite GFD being proposed as a therapeutic strategy for non-celiac autoimmune disorders [32]. This cautious approach stems from a recent review article about the possible nutritional deficiencies described in patients following a strict GFD, recently reviewed in [33]. However, because of the in vitro and in animal models’ evidence, this review highlights the need for more scientific evidence obtained by randomized trials regarding the relationship between a gluten-free diet and thyroid homeostasis.

## Figures and Tables

**Table 1:** Main characteristics of the studies.

First Author, Year [Ref]	Country	Design	Population	Case (*n*)	Control (*n*)
Metso, 2012 [14]	Finland	Prospective controlled study	Adult	27	27
Krysiak, 2019 [15]	Poland	Prospective non-randomized study	Adult	16	18
Asamoah, 2021 [16]	USA	Case report	Adult	1	0
Mainardi, 2002 [17]	Italy	Prospective study	Adult	2	0
Stramazzo, 2021 [18]	Italy	Letter to editor	Adult	1	0
Valentino, 1999 [19]	Italy	Prospective study	Adult	3	0

**Table 2:** Main findings of the included studies.

First Author [Ref]	Year	TSH Gluten-Free (mIU/L, Mean (SD))	TSH Normal Diet (mIU/L, Mean (SD))	fT3 Gluten-Free (pmol/L, Mean (SD))	fT3 Normal Diet (pmol/L, Mean (SD))	fT4 Gluten-Free (pmol/L, Mean (SD))	fT4 Normal Diet (pmol/L, Mean (SD))	Tg-Ab Gluten-Free (U/mL; Mean (SD))	Tg-Ab Normal Diet (U/mL; Mean (SD))	TPO-Ab Gluten-Free (U/mL; Mean (SD))	TPO-Ab Normal Diet (U/mL; Mean (SD))	25-Hydroxyvitamin D Gluten-Free (ng/mL, Mean (SD))	25-Hydroxyvitamin D Normal Diet (ng/mL, Mean (SD))
Krysiak [15]	2019	Baseline 2.7 (1.0), after 6 months 2.4 (0.8), change -0.3 (0.2)	Baseline 2.9 (0.8), after 6 months 2.6 (0.9), change -0.3 (0.2)	Baseline 3.2 (0.6), after 6 months 3.6 (0.7), change 0.4 (0.3)	Baseline 3.1 (0.6), after 6 months 3.2 (0.7), change 0.1 (0.3)	Baseline 14.9 (2.3), after 6 months 16.1 (2.4), change 1.2 (1.4)	Baseline 15.3 (2.7), after 6 months 15.0 (2.3), change 0.3 (0.6)	Baseline 832 (311), after 6 months 629 (240), change -203 (120)	Baseline 792 (274), after 6 months 845 (324), change 53 (58)	Baseline 925 (265), after 6 months 705 (206), change -200 (105)	Baseline 891 (242), after 6 months 920 (280), change 29 (25)	Baseline 20 (6), after 6 months 25 (6), change 5 (3)	Baseline 21 (5), after 6 months 20 (5), change -1 (2)
Metso [14]	2012	Median baseline 1.7, follow-up 12 months 1.7	Median baseline 1.5, follow-up 12 months 1.7	/	/	Median baseline 12.9 mU/L, follow-up 12 months 12.1 mU/L	Median baseline 12.1 mU/L, follow-up 12 months 12.7 mU/L	/	/	/	/	/	/
Asamoah [16]	2021	Baseline 3.57, after 3 months 0.77, after 12 months 0.45	/	/	/	Baseline 11.97, after 3 months 13.13, after 12 months 14.80	/	/	/	/	/	/	/
Mainardi [17]	2002	Baseline 13.5, after 10 months 7.65, after 18 months 0.65	/	Baseline 2.7 pg/mL, after 10 months 2.5	/	Baseline 0.85 ng/dL, after 10 months 0.86, after 18 months 1.18	/	Baseline 222, after 10 months 888, after 18 months 305	/	Baseline 265, after 10 months 716, after 18 months 289	/	/	/
Mainardi [17]	2002	Baseline 1.78, after 8 months 0.01	/	After 8 months 3.6 pg/ml	/	Baseline 0.73 ng/dL, after 8 months 1.4	/	Baseline 134, after 8 months 255	/	Baseline 474, after 8 months 586	/	/	/
Stramazzo [18]	2021	Baseline 3.5, after 3 months 3.98, after 6 months 3.45	/	/	/	Baseline 0.8 ng/dL, after 3 months 1, after 6 months 1.2	/	Baseline 756, after 3 months 1533	/	Baseline 549, after 3 months 651	/	/	/
Valentino [19]	1999	Baseline 9.8, after 6 months 1.5	/	/	/	Baseline 7.5 pg/mL, after 6 months 12	/	Baseline 212, after 6 months 206	/	Baseline 2580, after 6 months 1976	/	/	/
Valentino [19]	1999	Baseline 9.1, after 6 months 1.3	/	/	/	Baseline 8.2 pg/mL, after 6 months 10.9	/	Baseline 450, after 6 months 380	/	Baseline 1230, after 6 months 1115	/	/	/
Valentino [19]	1999	Baseline 7.9, after 6 months 1.1	/	/	/	Baseline 7.3 pg/mL, after 6 months 11.8	/	Baseline 312, after 6 months 210	/	Baseline 1580, after 6 months 1395	/	/	/

TSH: thyroid stimulating hormone; fT3: free T3; fT4: free T4; Tg-Ab: anti-thyroglobulin antibodies; TPO-Ab: anti-thyroid peroxidase antibodies; SD: standard deviation; unavailable data are indicated as /.

**Table 3:** Quality assessment of the studies according to QUADAS-2.

	Risk of Bias				Feasibility		
First Author	Patient’s Selection	Index Test	Reference Standard	Flow and Timing	Patient’s Selection	Index Test	Reference Standard
Krysiak	Low	Low	Low	Low	Low	Low	Low
Metso	Low	Unclear	Low	Low	Low	Low	Low
Mainardi	Low	Unclear	Unclear	Low	Low	Unclear	Low
Valentino	High	Unclear	Unclear	Low	Low	Unclear	Low

The studies by Asamoah [16] and Stramazzo [18] are not included in this quality assessment as they are a case report and letter to editor, respectively.

## Data Availability

Not applicable.

## Supplementary Materials

The following are available online at https://www.mdpi.com/article/10.3390/nu14081681/s1, Table S1: MOOSE (Meta-analyses Of Observational Studies in Epidemiology) Checklist.
