# Supplementary material for: What about TSH and Anti-Thyroid Antibodies in Patients with Autoimmune Thyroiditis and Celiac Disease Using a Gluten-Free Diet? A Systematic Review
Source: Nutrients. 2022 Apr 18;14(8):1681. doi: 10.3390/nu14081681 (PMC9028602; doi:10.3390/nu14081681)
Supplement: Supplementary file 1 [file nutrients-14-01681-s001.zip › nutrients-1662095-supplementary.pdf]

Supplementary Table S1: MOOSE (Meta-analyses Of Observational Studies in Epidemiology) Checklist

Item No	Recommendation	Page
Reporting of background should include		
1	Problem definition	3
2	Hypothesis statement	3
3	Description of study outcome(s)	3
4	Type of exposure or intervention used	3
5	Type of study designs used	3
6	Study population	3
Reporting of search strategy should include		
7	Qualifications of searchers (eg, librarians and investigators)	3
8	Search strategy, including time period included in the synthesis and key words	3
9	Effort to include all available studies, including contact with authors	3
10	Databases and registries searched	3
11	Search software used, name and version, including special features used (eg, explosion)	3
12	Use of hand searching (eg, reference lists of obtained articles)	3
13	List of citations located and those excluded, including justification	3
14	Method of addressing articles published in languages other than English	3
15	Method of handling abstracts and unpublished studies	3
16	Description of any contact with authors	3
Reporting of methods should include		
17	Description of relevance or appropriateness of studies assembled for assessing the hypothesis to be tested	3
18	Rationale for the selection and coding of data (eg, sound clinical principles or convenience)	3
19	Documentation of how data were classified and coded (eg, multiple raters, blinding and interrater reliability)	3
20	Assessment of confounding (eg, comparability of cases and controls in studies where appropriate)	3
21	Assessment of study quality, including blinding of quality assessors, stratification or regression on possible predictors of study results	3
22	Assessment of heterogeneity	3
23	Description of statistical methods (eg, complete description of fixed or random effects models, justification of whether the chosen models account for predictors of study results, dose-response models, or cumulative meta-analysis) in sufficient detail to be replicated	3
24	Provision of appropriate tables and graphics	3
Reporting of results should include		
25	Graphic summarizing individual study estimates and overall estimate	4
26	Table giving descriptive information for each study included	4
27	Results of sensitivity testing (eg, subgroup analysis)	4
28	Indication of statistical uncertainty of findings	4
Reporting of discussion should include		
29	Quantitative assessment of bias (eg, publication bias)	4
30	Justification for exclusion (eg, exclusion of non-English language citations)	4
31	Assessment of quality of included studies	4
Reporting of conclusions should include		
32	Consideration of alternative explanations for observed results	4-5
33	Generalization of the conclusions (ie, appropriate for the data presented and within the domain of the literature review)	4-5
34	Guidelines for future research	5
35	Disclosure of funding source	1

From: Stroup DF, Berlin JA, Morton SC, et al, for the Meta-analysis Of Observational Studies in Epidemiology (MOOSE) Group. Meta-analysis of Observational Studies in Epidemiology. A Proposal for Reporting. *JAMA*. 2000;283(15):2008-2012. doi: 10.1001/jama.283.15.2008.
